# Predictors of lung function decline in scleroderma-related interstitial lung disease based on high-resolution computed tomography: implications for cohort enrichment in systemic sclerosis–associated interstitial lung disease trials

**DOI:** 10.1186/s13075-015-0872-2

**Published:** 2015-12-23

**Authors:** Dinesh Khanna, Vivek Nagaraja, Chi-hong Tseng, Fereidoun Abtin, Robert Suh, Grace Kim, Athol Wells, Daniel E. Furst, Philip J. Clements, Michael D. Roth, Donald P. Tashkin, Jonathan Goldin

**Affiliations:** University of Michigan Scleroderma Program, Division of Rheumatology, Department of Internal Medicine, University of Michigan, Suite 7C27, 300 North Ingalls Street, SPC 5422, Ann Arbor, MI 48109 USA; Division of Rheumatology, University of Toledo, Toledo, OH USA; Department of Biostatistics, David Geffen School of Medicine at UCLA, Los Angeles, CA USA; Department of Radiology, David Geffen School of Medicine at UCLA, Los Angeles, CA USA; Division of Pulmonary and Critical Care, Royal Brompton Hospital, London, UK; Department of Medicine, David Geffen School of Medicine at UCLA, Los Angeles, CA USA

**Keywords:** Systemic sclerosis, Interstitial lung disease, High-resolution computed tomography, Cohort enrichment, Randomized controlled trial, Scleroderma lung disease, Scleroderma Lung Study I (SLS I), Goh and Wells criteria

## Abstract

**Background:**

The extent of lung involvement visualized by high-resolution computed tomography (HRCT) is a predictor of decline in forced vital capacity (FVC) in scleroderma–interstitial lung disease. Our objective was to evaluate the performance of three different HRCT-defined staging systems in the Scleroderma Lung Study I (SLS I) over a 1-year period.

**Methods:**

We assessed two visual semiquantitative scores: the maximum fibrosis score (MaxFib, the fibrosis score in the zone of maximal lung involvement) and visual assessment of total lung involvement (TLI) as proposed by Goh and Wells. In addition, we evaluated the computer-aided diagnosis and calculated the quantitative percentage with fibrosis (QLF) and TLI.

**Results:**

The mean duration of the disease was 3.2 years, and the mean FVC was 67.7 %. Regardless of the staging system used, a greater degree of fibrosis/TLI on HRCT scans was associated with a greater decline in FVC in the placebo group. Using the MaxFib and QLF, the mean absolute changes in FVC from baseline were 0.1 % and −1.4 %, respectively, in <25 % lung involvement vs. a change of −6.2 % and −6.9 %, respectively, with >25 % involvement (negative score denotes worsening in FVC). Conversely, cyclophosphamide was able to stabilize decline in FVC in subjects with greater degree of involvement detected by HRCT. Using the visual MaxFib and QLF, the mean absolute improvements in FVC were 1.2 and 1.1, respectively, with >25 % involvement.

**Conclusions:**

HRCT-defined lung involvement was a predictor of decline in FVC in SLS I. The choice of staging system for cohort enrichment in a clinical trial depends on feasibility.

**Trial registration:**

ClinicalTrials.gov identifier: NCT00004563 (Scleroderma Lung Study I)

ISRCTN15982171. Registered 19 Aug 2015.

**Electronic supplementary material:**

The online version of this article (doi:10.1186/s13075-015-0872-2) contains supplementary material, which is available to authorized users.

## Background

Systemic sclerosis–associated interstitial lung disease (SSc-ILD) is common, is associated with a poor prognosis, and is the leading cause of death in people with SSc [1]. The pathogenesis of SSc-ILD involves a complex interplay of vascular injury, inflammation, and fibrosis (reviewed in [2–6]). The most common pathological finding in lung biopsies of patients with SSc-ILD (approximately 78 % patients) is nonspecific interstitial pneumonia [7]. Usual interstitial pneumonia, the pathological finding in idiopathic pulmonary fibrosis (IPF), as well as other patterns are present in approximately 10–15 % of patients with SSc-ILD [7]. However, open lung biopsy is usually not performed for SSc-ILD, and high-resolution computed tomography (HRCT) has become the gold standard for diagnosis and classification of ILD [8, 9]. In addition to diagnosis of ILD, moderate to severe fibrosis or total lung involvement (TLI) by SSc-ILD visualized on the baseline HRCT scan is an independent predictor of response to cyclophosphamide (CYC) therapy [10], poor survival [11], and future decline in percentage of predicted forced vital capacity (FVC% predicted) [12].

Recent trials have included forced vital capacity (FVC), which has traditionally served as the primary endpoint in SSc-ILD clinical trials as it is available. It has low measurement error (if done using standardized methodology) and is sensitive to change in treatment. However, treatment with CYC had only modest effects on FVC in the Scleroderma Lung Study I (SLS I) [13, 14] and the Fibrosing Alveolitis in Scleroderma Trial (FAST) [15]. Therefore, there is increased interest in enriching this cohort of patients with rapid disease progression for effective identification of patients at high risk of ILD progression, as well as for early intervention [16].

Different HRCT staging systems have been developed to quantify the extent of lung involvement–semiquantification by visual assessment or quantification using computer-assisted methodology. Kazerooni et al. developed a semiquantitative measure to assess ground-glass opacity (GGO), reticulations with architectural distortion and traction bronchiectasis (“fibrosis”), and honeycomb cysts (HCs) [17], and the overall score correlated well with assessment of ILD in pathological specimens [17]. A modified Kazerooni visual scoring system was used for SLS I, a placebo-controlled trial of oral CYC in patients with symptomatic SSc-ILD in which the extent of reticulations (“fibrosis”), GGO, and HCs was scored semiquantitatively. In addition, a novel algorithm was developed to quantify the presence and extent of both fibrosis and total ILD (sum of scores for fibrosis, GGO, and HCs) using the computer-aided diagnosis (CAD) technology in three area-equivalent zones (upper, middle, and lower) as well as in the whole lung (WL) in SLS I [14, 18, 19]. CAD is based upon measurement of the density or texture features of each pixel and assignment of a score for the amount of abnormal lung tissue present. Quantitative assessment of total extent of interstitial lung disease (QILD) and of lung fibrosis (QLF) correlates well with the visual scoring systems and provides an objective determination of treatment efficacy in patients with SSc-ILD [20] (Additional file 1: Figure S1). Separately, Goh and Wells developed and validated a visual semiquantitative staging system for TLI (i.e., fibrosis, GGO, and HCs) in an observational cohort of patients with SSc-ILD [11].

We used individual patient data from the SLS I for current analysis. SLS I was a multicenter, double-blind, randomized controlled trial (RCT) conducted to evaluate the effectiveness and safety of oral CYC administered for 1 year in patients with symptomatic SSc-ILD who had evidence of ILD. The SLS I was the first RCT to demonstrate the effectiveness of CYC in FVC, relative to placebo, at the end of the 1-year treatment period [14]. Although the physiological benefits of CYC compared with placebo were modest (2.53 % and 4.09 % improvements in FVC% predicted and total lung capacity, respectively, at 12 months; *p* < 0.03), these results were supported by parallel findings of improvement in patient-reported outcomes (health-related quality of life, cough, and dyspnea) [21, 22], as well as greater stability of fibrosis visualized by HRCT ([23–25] and summarized in [16]) and skin thickness scores. In addition, follow-up HRCT scans obtained at 12 months revealed that the change in extent of fibrosis from baseline was significantly worse in the placebo group than in the CYC treatment group [25]. Our objective was to compare the performance in a post hoc analysis of different HRCT staging systems on FVC and diffusing capacity for carbon monoxide (DLCO) in SLS I over a 1-year period. Specifically, we sought to determine (1) whether an HRCT staging system can enrich for subjects who will most likely decline in the placebo group and (2) the effects of HRCT staging system on the expected changes in FVC and DLCO in the CYC group that may inform the design of future trials. We chose a 1-year period on the basis of expert consensus that SSc-ILD trials should be at least 1 year [16].

## Methods

### Patient population

SLS I consisted of 158 participants randomized to receive either oral CYC or a matching placebo for 1 year, followed by an additional year of observation off treatment, as previously published [14]. Ethical approval was received from each participating institution and written informed consent was obtained from each subject. Briefly, inclusion criteria included age ≥18 years, duration of disease ≤7 years from onset of the first non-Raynaud’s symptom of SSc, FVC% 40–85 %, DLCO ≥40 % predicted (or 30–39 % predicted in the absence of clinical evidence of pulmonary hypertension), and evidence of any GGO and/or positive bronchoalveolar lavage (≥3 % neutrophils and/or ≥2 % eosinophils). All subjects provided written informed consent, and the study was approved by the medical institutional review board at each clinical center. Please see the Acknowledgments section for the list of centers that participated in the study.

### Baseline measurements

Baseline measurements included full pulmonary function tests (PFTs), including spirometry, lung volume (by body plethysmography), and DLCO. PFTs were read centrally for quality assurance. In addition to ascertainment of disease duration and presence of limited or diffuse cutaneous SSc, patient-centered measures (including dyspnea and quality-of-life indices) were obtained. HRCT scans were obtained at baseline with the patient in prone position and at maximal inspiration. The images were acquired from scanners with at least four multidetectors according to a standardized protocol. Nonvolumetric computed tomographic scans of –2-mm slice thickness acquired at 10-mm increments were acquired contiguously. More details are available elsewhere [24].

### HRCT staging systems

In SLS I, HRCT scans were scored by two independent radiologists who used a Likert scale (0 = absent, 1 = 1–25 %, 2 = 26–50 %, 3 = 51–75 %, and 4 = 76–100 %) for extent of four categories of parenchymal abnormality (pure GGO), lung fibrosis, HCs, and emphysema) [14]. The scoring was performed for each of the three zones (upper, extending from apex to aortic arch; middle, from aortic arch to inferior pulmonary vein; and lower, from inferior pulmonary veins to base) in each lung, as well as for the WL. The visual maximum fibrosis score (MaxFib) was individually reviewed by two thoracic radiologists who scored fibrosis in the zone of maximal involvement (ZM). Discordant interpretations were reviewed with a third radiologist to achieve consensus in a face-to-face meeting; no average scores were calculated. Quantitative maximum extent of fibrosis was also determined by CAD in the ZM.

### Goh and Wells system

Goh and Wells developed a prognostic algorithm for patients with SSc-ILD, integrating PFTs and HRCT [11]. TLI was assessed in an observational cohort of patients with SSc-ILD. HRCT images were scored by two independent radiologists at five levels: (1) origin of great vessels, (2) main carina, (3) pulmonary venous confluence, (4) halfway between the third and fifth sections, and (5) immediately above the right hemidiaphragm. HRCT variables were total disease extent that incorporated the extent of a reticular pattern, GGO, and HCs. The extent of ILD was estimated as a percentage of total volume to the nearest 5 % in each of the five sections. Global extent of disease determined by HRCT was calculated as the mean extent score in the five scored sections. This was stratified as <20 % vs. >20 % TLI (termed *Goh and Wells unadjusted stratification*). For indeterminate cases (extent of TLI 10–30 % because there may be measurement error by visual read), an FVC threshold of 70 % is an adequate prognostic substitute. On the basis of these observations, Goh and Wells staged global extent of disease as limited or minimal disease (minimal disease determined by HRCT or, in indeterminate cases, FVC ≥70 %) or extensive disease (severe disease determined by HRCT or, in indeterminate cases, FVC <70 %) [11]. This aspect has been termed *Goh and Wells adjusted stratification*.

### Computer-aided diagnosis

To obviate interreader variation and to standardize data across multiple sites, a CAD was developed using the SLS I data [18]. The HRCT scans from SLS I were reconstructed with sharp or manufacturer-recommended overenhancing filters. The CAD system segmented each lung of each patient into three area-equivalent zones (upper, middle, and lower). After semiautomated lung segmentation, the images were entered into a quantitative image workstation to produce separate quantitative scores for reticulations (fibrosis), GGO, and HCs automatically [19]. The QILD score was the sum of all abnormally classified scores, including scores for fibrosis, GGO, and HCs. HRCT QLF scores were determined using the percentage of counts in which the classified abnormal pattern comprised reticular opacity with architectural distortion. QILD and QLF scores were summed for both the WL, including both lungs, and the ZM.

A 25 % threshold for the QILD and QLF in the ZM was agreed a priori to be consistent with a visual MaxFib cutoff of 25 % (which assesses area of maximum fibrosis). In addition, we agreed to a 20 % cutoff for QILD and QLF in the WL to be consistent with a Goh and Wells threshold of 20 % for TLI.

### Statistical analysis

We analyzed HRCT data in two different ways. First, we assessed the strength of associations between each staging system and FVC or DLCO percentage of predicted value at baseline, as well as the change in FVC and DLCO percentage of predicted value from baseline to 12 months using Pearson correlation coefficients. A coefficient ≥0.40 was considered to be a moderate association [26]. Second, we stratified the staging system (e.g., 0–25 % vs. 26–100 % for visual MaxFib) on the basis of published data to assess if this could enrich for subjects in the placebo group who would decline over a 1-year period. Two-sample *t* tests were used to assess statistical significance for absolute and relative change (percentage of predicted change from baseline) in FVC and DLCO over a 1-year period. Fisher’s exact test or a χ^2^ test was used to compare categorical variables among categories within the different staging systems. All tests were two-sided, and a *p* value <0.05 was considered statistically significant. All analyses were performed using SAS 9.3 software (SAS Institute, Cary, NC, USA).

## Results

Of the 158 patients, 93 (48 patients in the placebo group and 45 patients in the CYC group) had FVC data available at baseline and 12 months as well as good-quality baseline HRCT scans. These patients were included in the analysis. There were no significant differences in baseline characteristics between the two treatment groups (Table 1). These patients’ mean age was 47 years, and their mean disease duration was 3.2 years. Their mean (standard deviation [SD]) FVC was 67.7 % (11.9) of predicted, and their mean (SD) DLCO was 46.3 % (12.7) of predicted.

**Table 1:** Baseline patient characteristics, stratified by disease duration

Variable	All patients (*n* = 93)	Placebo (*n* = 48)	CYC (*n* = 45)	*p* Value
Age, yr, mean (SD)	47.19 (11.72)	47.43 (13.24)	46.93 (10.00)	0.8388
Female sex, *n* (%)	68 (73.12)	34 (70.83)	34 (75.56)	0.6077
White race, *n* (%)	71 (76.34)	38 (79.17)	33 (73.33)	0.6764
Type of SSc, *n* (%)				
Limited	37 (39.78)	19 (39.58)	18 (40.00)	0.9673
Diffuse	56 (60.22)	29 (60.42)	27 (60.00)	
Disease duration, yr, mean (SD)	3.27 (2.24)	3.30 (1.97)	3.24 (2.52)	0.8895
Antibodies (*n* = 55), *n* (%)^a^				
Scl-70	22 (32.84)	13 (35.14)	9 (30.00)	0.6563
Anti-centromere/anti-RNA polymerase III	14 (20.90)	5 (13.51)	9 (30.00)	0.0988
FVC, % predicted, mean (SD)	67.73 (11.90)	68.86 (11.91)	66.53 (11.90)	0.3497
DLCO, % predicted, mean (SD)	46.32 (12.75)	46.04 (12.41)	46.61 (13.24)	0.8303
MRSS, mean (SD)	15.22 (11.02)	14.56 (10.52)	15.91 (11.60)	0.5580
Mahler’s BDI focal score (0–12), mean (SD)	5.67 (1.76)	5.48 (1.99)	5.90 (1.45)	0.2613
HAQ-DI (0–3), mean (SD)	0.82 (0.67)	0.70 (0.67)	0.96 (0.64)	0.0629
SF-36 PCS (0–100), mean (SD)	33.70 (11.29)	34.63 (10.98)	32.68 (11.66)	0.4099
SF-36 MCS (0–100), mean (SD)	49.15 (10.94)	49.23 (11.17)	49.06 (10.82)	0.9413
HRCT-determined disease extent, mean (SD)				
Maximum fibrosis score (0–4)	1.99 (1.05)	1.96 (1.07)	2.02 (1.03)	0.7700
Maximum honeycombing (0–4)	0.40 (0.58)	0.42 (0.54)	0.37 (0.62)	0.9592
Maximum ground-glass opacity (0–4)	0.73 (0.76)	0.73 (0.71)	0.72 (0.83)	0.7101
Visual maximum fibrosis score, *n* (%)				0.2007
0 %	7 (7.69)	4 (8.33)	3 (6.98)	
1–25 %	25 (27.47)	15 (31.25)	10 (23.26)	
26–50 %	25 (27.47)	9 (18.75)	16 (37.21)	
51–75 %	30 (32.97)	19 (39.58)	11 (25.58)	
76–100 %	4 (4.40)	1 (2.08)	3 (6.98)	
Goh and Wells unadjusted stratification for lung involvement (consensus of 3 readers), *n* (%)				0.5298
<20 %	38 (44.71)	22 (47.83)	16 (41.03)	
>20 %	47 (55.29)	24 (52.17)	23 (58.97)	
Goh’s adjusted minimal disease, *n* (%)	20 (21.98)	8 (17.02)	12 (27.27)	0.3540
Goh’s adjusted extensive disease, *n* (%)	71 (78.02)	39 (82.98)	32 (72.73)	
CAD scores				
QILD WL, mean (SD)	34.71 (15.67)	34.96 (16.95)	34.44 (14.35)	0.8771
QILD ZM, mean (SD)	58.84 (20.94)	58.53 (21.33)	59.19 (20.75)	0.8833
QLF WL, mean (SD)	9.80 (9.83)	10.11 (10.60)	9.47 (9.02)	0.7591
QLF ZM, mean (SD)	26.44 (21.82)	25.23 (21.44)	27.77 (22.40)	0.5851

*FVC* forced vital capacity, *DLCO* diffusing capacity for carbon monoxide, *MRSS* modified Rodnan skin thickness score, *BDI* Baseline Dyspnea Index, *HAQ-DI* Health Assessment Questionnaire Disability Index, *PCS* Physical Component Score, *SF-36* 36-item Short Form Health Survey, *MCS* Mental Component Score, *HRCT* high-resolution computed tomography, *CAD* computer-aided diagnosis, *QILD* quantitative assessment of total extent of interstitial lung disease, *QLF* quantitative percentage of lung fibrosis, *ZM* zone of maximal involvement, *WL* whole lung, *SD* standard deviation, *SSc* systemic sclerosis, *CYC* cyclophosphamide

^a^ *p* < 0.05

First, we assessed the relationships between the scoring systems as continuous variables and change in FVC and DLCO. In the placebo group, when absolute changes in FVC and DLCO were considered, correlations between the staging systems were largely negative but none were significant (Table 2). Conversely, these correlations were positive in the CYC group, suggesting that CYC treatment had a positive impact on FVC and DLCO in patients with a greater degree of ILD (assessed using different staging systems).

**Table 2:** Correlation coefficients between the staging systems vs. the PFT parameters (FVC and DLCO at baseline and after 12 months of treatment)

Staging systems	FVC (baseline)		FVC (absolute change)		DLCO (baseline)		DLCO (absolute change)	
	Placebo	CYC	Placebo	CYC	Placebo	CYC	Placebo	CYC
MaxFib	−0.21 (0.15)	−0.16 (0.29)	−0.31 (0.88)	0.34 (0.02)	−0.46 (0.001)	−0.44 (0.003)	−0.02 (0.88)	0.13 (0.41)
Goh and Wells unadjusted stratification	−0.05 (0.75)	−0.25 (0.09)	−0.21 (0.15)	0.10 (0.50)	−0.48 (0.001)	−0.51 (0.001)	0.003 (0.98)	0.17 (0.27)
QILD WL	−0.38 (0.008)	−0.08 (0.61)	−0.23 (0.12)	0.41 (0.006)	−0.35 (0.01)	−0.07 (0.63)	−0.35 (0.02)	0.12 (0.45)
QILD ZM	−0.27 (0.07)	−0.19 (0.23)	−0.20 (0.17)	0.40 (0.008)	−0.41 (0.005)	−0.24 (0.12)	−0.30 (0.04)	0.25 (0.10)
QLF WL	−0.17 (0.26)	−0.25 (0.11)	−0.22 (0.13)	0.06 (0.62)	−0.22 (0.13)	−0.20 (0.20)	−0.10 (0.50)	0.16 (0.32)
QLF ZM	−0.45 (0.002)	−0.39 (0.02)	−0.15 (0.31)	0.18 (0.23)	−0.43 (0.002)	−0.41 (0.005)	−0.22 (0.13)	0.12 (0.45)

*FVC* forced vital capacity, *MaxFib* visual maximum fibrosis score, *QILD WL* quantitative assessment of total extent of interstitial lung disease in whole lung, *QILD ZM* quantitative assessment of total extent of interstitial lung disease in zone of maximal involvement, *QLF WL* quantitative percentage with fibrosis in whole lung, *QLF ZM* quantitative percentage with fibrosis in zone of maximal involvement, *CYC* cyclophosphamide, *DLCO* diffusing capacity for carbon monoxide

The values are Pearson’s correlation coefficients; *p* values are presented in parentheses

**Fig. 1:**
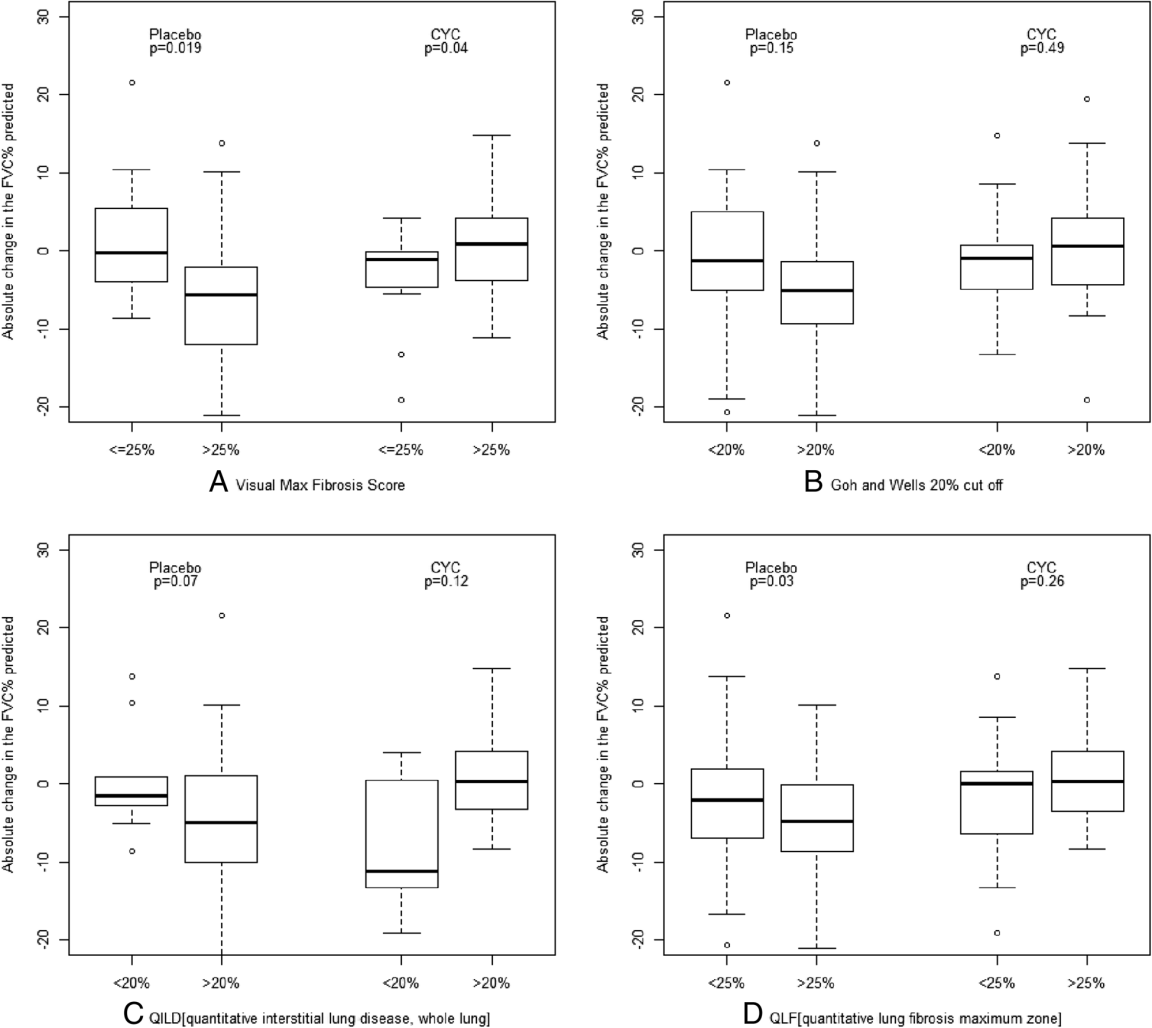
Absolute changes in percentage of predicted forced vital capacity (FVC%) determined using different high-resolution computed tomography (HRCT) staging systems. Data are shown as box plots. Each box represents the interquartile range (IQR), indicating the first (25th percentile) and third (75th percentile) quartiles. *Lines* inside the boxes represent the medians. *Whiskers* represent 1.5 times the upper and lower IQRs. *Circles* indicate outliers. *p* Value is based on two-samples *t* test. **a** Visual semiquantitative fibrosis score. **b** Goh and Wells unadjusted stratification. **c** Quantitative assessment of total extent of interstitial lung disease (QILD) in whole lung. **d** Quantitative percentage with fibrosis (QLF) in zone of maximal involvement

Next, we assessed whether various scoring systems, when dichotomized into mild vs. extensive disease, could predict change in PFTs. The absolute decline in FVC based on different staging systems is shown in Table 3 and Fig. 1. In the placebo group, regardless of the staging system used, there was a decline in FVC in patients with more extensive disease. For example, for MaxFib, the mean (SD) percentage decline in FVC was −6.2 (12.5) with >25 % involvement and 0.1 (9.0) (mild improvement) with <25 % involvement (*p* = 0.01). When we used the Goh and Wells unadjusted stratification for extent of ILD involvement, the mean (SD) declines in FVC were −1.6 (10.2) with <20 % lung involvement and −5.5 (8.0) with >20 % involvement. Similar trends were observed in the CAD staging system. When we used QILD for WL involvement, patients with >20 % involvement had a decline of −4.9 (9.5) vs. improvement of 0.3 (6.8) for <20 % involvement. Similar trends were also seen when change from baseline was expressed as relative decline in FVC percentage of predicted value (Additional file 2: Table S1).

**Table 3:** Absolute decline in FVC percentage of predicted value (compared with baseline) over 12 months

	Placebo group			Cyclophosphamide group		
	Number of subjects	Absolute decline in FVC % predicted, mean (SD)	*p* Value	Number of subjects	Absolute decline in FVC % predicted, mean (SD)	*p* Value
Visual maximum fibrosis score						
0–25 %	19	0.1 (9.0)	0.019	13	−3.4 (6.3)	0.04
26–100 %	29	−6.2 (8.3)		30	1.2 (6.6)	
Goh and Wells criteria, unadjusted stratification						
<20 %	22	−1.6 (10.2)	0.15	15	−1.0 (6.7)	0.49
>20 %	25	−5.5 (8.0)		30	0.6 (7.7)	
Goh and Wells criteria with indeterminate results on HRCT (10–30 %), adjusted stratification						
Minimal disease	8	2.2 (9.8)	0.08	12	−1.1 (4.3)	0.47
Extensive disease	39	−4.9 (8.7)		32	0.3 (8.2)	
QILD WL						
<20 %	10	0.3 (6.8)	0.07	5	−7.9 (9.7)	0.12
>20 %	37	−4.9 (9.5)		38	0.8 (5.7)	
QILD ZM						
<25 %	2	5.0 (7.5)	0.32	2	−12.3 (1.5)	0.001
>25 %	45	−4.1 (9.1)		41	0.4 (6.4)	
QLF WL						
<20 %	42	−3.0 (9.1)	0.16	39	−0.4 (6.7)	0.70
>20 %	5	−9.8 (8.7)		4	1.4 (8.2)	
QLF ZM						
<25 %	27	−1.4 (10.1)	0.03	24	−1.2 (7.2)	0.26
>25 %	20	−6.9 (6.8)		19	1.1 (6.2)	

*FVC % predicted* percentage of predicted forced vital capacity, *HRCT* high-resolution computed tomography, *QILD WL* quantitative assessment of total extent of interstitial lung disease in whole lung, *QILD ZM* quantitative assessment of total extent of interstitial lung disease in zone of maximal involvement, *QLF WL* quantitative percentage with fibrosis in whole lung, *QLF ZM* quantitative percentage with fibrosis in zone of maximal involvement, *SD* standard deviation Negative score denotes worsening in FVC

In the CYC arm, nonsignificant changes in FVC were noted across the various staging systems, most notably in patients with greater involvement visualized by HRCT (Table 3 and Additional file 2: Table S1). In the MaxFib group, a small improvement in FVC was seen in patients with >25 % involvement (1.2 [6.6]; *p* = 0.04). Although not statistically significant, similar changes were seen in the Goh and Wells unadjusted stratification, the Goh and Wells adjusted stratification, and the CAD staging systems.

The absolute decline in DLCO in relation to the choice of staging systems is shown in Table 4. In the placebo group, a statistically significant difference in DLCO was observed in QILD-WL staging by CAD (<20 %, 4.8 [8.6]; >20 %, −4.3 [10.6]; *p* = 0.01). Although statistical significance was not reached in the other staging systems, the mean changes in DLCO showed a trend toward a significant difference in patients with greater HRCT involvement in the placebo group. A larger variability was noted in the CYC arm, with a statistically significant effect on Goh and Wells adjusted stratification (minimal −1.3 [7.9] vs. extensive −6.9 [6.9]; *p* = 0.4) (Table 4).

**Table 4:** Absolute decline in DLCO from baseline over 12 months

	Placebo group			Cyclophosphamide group		
	Number of subjects	Absolute decline in DLCO from baseline (%), mean (SD)	*p* Value	Number of subjects	Absolute decline in DLCO from baseline (%), mean (SD)	*p* Value
Visual maximum fibrosis score						
0–25 %	19	−1.6 (10.8)	0.62	13	−6.0 (9.2)	0.64
26–100 %	29	−3.2 (10.9)		30	−4.7 (7.2)	
Goh and Wells criteria, unadjusted stratification						
<20 %	22	−3.0 (11.5)	0.98	15	−6.9 (8.4)	0.30
>20 %	25	−3.0 (9.9)		30	−4.3 (7.1)	
Goh and Wells criteria with indeterminate results on HRCT (10–30 %), adjusted stratification						
Minimal disease	8	−1.65 (11.5)	0.84	12	−1.3 (7.9)	0.04
Extensive disease	39	−2.5 (10.8)		32	−6.9 (6.9)	
QILD WL						
<20 %	10	4.8 (8.6)	0.01	5	−7.6 (10.2)	0.58
>20 %	37	−4.3 (10.6)		38	−4.7 (7.4)	
QILD ZM						
<25 %	2	12.7 (3.2)	0.02	2	−13.7 (3.0)	0.08
>25 %	45	−3.0 (10.5)		41	−4.7 (7.7)	
QLF WL						
<20 %	42	−1.9 (10.4)	0.63	39	−5.5 (7.9)	0.08
>20 %	5	−5.9 (14.9)		4	−1.4 (3.2)	
QLF ZM						
<25 %	21	0.3 (12.3)	0.15	20	−6.0 (8.1)	0.46
>25 %	26	−4.5 (9.1)		23	−4.2 (7.5)	

*DLCO* diffusing capacity for carbon monoxide, *HRCT* high-resolution computed tomography, *QILD WL* quantitative assessment of total extent of interstitial lung disease in whole lung, *QILD ZM* quantitative assessment of total extent of interstitial lung disease in zone of maximal involvement, *QLF WL* quantitative percentage with fibrosis in whole lung, *QLF ZM* quantitative percentage with fibrosis in zone of maximal involvement, *SD* standard deviation

## Discussion

As somewhat effective therapies for other manifestations of SSc (e.g., renal, pulmonary arterial hypertension, and articular) have emerged [27, 28], the morbidity and mortality of ILD have become increasingly apparent [29–31]. Traditionally, the severity of SSc-ILD is defined by the degree of ventilatory restriction in conjunction with the magnitude of diffusion impairment. These physiological measures are indirect and highly variable surrogates for the extent of structural disease abnormality [32]. In contrast, the extent of ILD visualized on HRCT images (fibrosis, GGO, and/or HCs) is a more direct and precise indicator of the severity of the underlying pathological process [10–12, 20] and is associated with mortality [3].

With increasing interest in optimizing the design of clinical trials for evaluation of interventions for SSc-ILD, it is important to reliably identify cohorts of patients with a higher risk of disease progression and a greater likelihood of a favorable response to disease-modifying therapy. This process of cohort enrichment consists of the selective enrollment of these patients in treatment studies, reducing the patient numbers required to demonstrate a treatment effect, and increasing the average amplitude of such a benefit [16]. Our group previously published post hoc multivariate regression analyses [10] using the SLS I and identified that fibrosis at baseline determined by HRCT, the modified Rodnan skin thickness score (MRSS), and the Mahler Baseline Dyspnea Index as independent correlates of treatment response to CYC. When patients were stratified on the basis of whether 50 % or more of any lung zone was involved by reticular infiltrates in the ZM as determined by HRCT, as assessed by visual scoring, and/or whether patients exhibited an MRSS ≥23 (0–51 scale), a subgroup of patients emerged in whom there was an average CYC treatment effect of 9.81 % at 18 months (*p* < 0.001). Conversely, there was no treatment effect (−0.58 % difference) in patients with less severe HRCT findings and a lower MRSS at baseline.

The present study represents another step toward defining cohort enrichment for clinical trials. We compared three different staging systems used to quantify the extent of ILD on HRCT: the visual MaxFib score, the Goh and Wells criteria, and the CAD quantitative scores for fibrosis (QLF) and TLI (QILD). In the placebo group, patients categorized as having moderate to extensive ILD on the basis of any of the three staging systems had a larger absolute decline in FVC (Table 3). Although MaxFib had the highest statistical significance for the placebo group, the differences in absolute changes in FVC between different staging systems were small and had similar trends in decline of FVC with greater HRCT involvement. In the CYC arm, there was stabilization in FVC in patients with extensive disease visualized by HRCT across all the staging systems. Interestingly, higher HRCT-assessed involvement was associated with stabilization of FVC in the CYC group vs. average decline in the other HRCT group. Both MaxFib and QILD-ZM showed statistically significant changes. This is consistent with correlation coefficient data where there are positive correlations with change in FVC vs. different staging systems in the CYC group (Table 2). We also included detailed analysis for the CYC group based on preliminary data from the SLS II, a double-blind study of mycophenolate mofetil vs. CYC in patients with SSc with symptomatic ILD treated with oral mycophenolate mofetil for 2 years compared with oral CYC for 1 year followed by placebo during the second year [33]. Interestingly, it appears that background CYC therapy negates the enrichment strategy using HRCT staging systems. The change in FVC was positive (suggesting stabilization and/or improvement) in the more severe HRCT lung involvement with all staging systems. This analysis can inform trial design in future studies in which researchers consider background immunosuppressive therapies.

FVC was used as the primary outcome measure in the SLS I and FAST studies. The treatment with CYC had only a modest effect on FVC in the SLS I [13, 14] and FAST trials, and clinicians have debated the meaning of these results in clinical care [34, 35]. On the basis of a recent viewpoint published by the U.S. Food and Drug Administration on FVC in IPF [36], we explored whether cohort enrichment in the SLS I population could have provided a more clinically meaningful change in FVC compared with the entire sample. Although not established for SSc-ILD, a change of 2–6 % is considered a minimally important change in IPF [37]. Using different staging systems, we found that patients with extensive lung involvement determined by HRCT had clinically meaningful declines.

Using the SLS I and II, we recently showed that DLCO is the single best correlate of the extent of lung involvement determined by HRCT [32] and supported by the correlation coefficients between different staging systems vs. baseline DLCO (Table 3). However, DLCO has the high measurement error and lack of specificity (as it is influenced by both ILD and pulmonary vascular disease [16, 38]), and none of the staging systems were correlated with the change in DLCO over 1 year, highlighting that DLCO is a poor outcome measure in ILD trials.

Our analysis may have significant impact on clinical trial design. This information can be used to enrich patients who are recruited in future ILD trials, calculate sample size, and judge the feasibility of the trial. For example, using visual MaxFib score,  73 % of patients who participated in the SLS I would qualify for an enriched protocol. Although this analysis does not provide guidance regarding which staging system to incorporate, recent post hoc analyses from SLS I suggest that the CAD system is more sensitive to change than a visual scoring system [19, 23, 25, 32]. Therefore, if HRCT is planned as an outcome measure (in addition to an enrichment criterion), then CAD is the preferred system [19, 23, 24], depending on its availability. Also, Goh and Wells criteria are applied only to the baseline HRCT and have not been evaluated in a longitudinal fashion. However, they have the advantage that they can easily be incorporated into observational studies [39]. Conversely, the CAD system is not universally available, which may limit its feasibility.

Our study has much strength in its comparison of three staging systems that have been published for grading the extent of SSc-ILD and have been shown to be feasible for use in a clinical trial. In addition, we validated the Goh and Wells criteria in SLS I.

Our study is not without limitations. The analysis is a post hoc analysis and is limited to participants enrolled in a clinical trial with specific entry criteria, thereby limiting the generalizability of the findings. The number of subjects in the study is low, and further validation is needed in another cohort to confirm the results. Use of the staging system in other cohorts (including clinical trials and observational cohorts) should be carefully assessed before the findings are generalized.

## Conclusions

The extent of HRCT-quantified ILD is a predictor of decline in FVC over a 1-year period and is independent of the staging system used to classify extent of disease. The choice of the staging system in a clinical trial depends on feasibility and available expertise but should be validated before incorporating it in future studies.

## Data-sharing statement

Anonymized data from SLS I are available to investigators by application to the SLS I Executive Committee (DPT: dtashkin@ mednet.ucla.edu).

## Additional files

Additional file 1: Figure S1. Representative data from a subject at baseline and 12 months in the SLS I. Subject was 61 years old with baseline and 12-month HRCT. Goh and Wells unadjusted stratification >20 (**a** and **b**). In whole lung, quantitative lung fibrosis scores (*blue dots*) are 9.71 % at baseline (**c**) and 34.25 % at 12 months (**d**); quantitative ground glass (*yellow dots*) are 18.38 % at baseline and 25.8 % at 12 months, and QILD score are 38.09 % at baseline and 60.05 % at 12-month follow-up. (PPTX 945 kb) Additional file 2: Table S1. Relative decline in FVC % predicted (compared with baseline) over 12 months (DOCX 15 kb)

## Abbreviations

BDI: Baseline Dyspnea Index
CAD: computer-aided diagnosis
CYC: cyclophosphamide
DLCO: diffusing capacity for carbon monoxide
FAST: Fibrosing Alveolitis in Scleroderma Trial
FVC: forced vital capacity
FVC% predicted: percentage of predicted forced vital capacity
GGO: ground-glass opacity
HAQ-DI: Health Assessment Questionnaire Disability Index
HC: honeycomb cyst
HRCT: high-resolution computed tomography
IPF: idiopathic pulmonary fibrosis
IQR: interquartile range
MaxFib: maximum fibrosis score
MCS: Mental Component Score
MRSS: modified Rodnan skin thickness score
PCS: Physical Component Score
PFT: pulmonary function test
QILD: quantitative assessment of total extent of interstitial lung disease
QLF: quantitative percentage with fibrosis
RCT: randomized controlled trial
SD: standard deviation
SF-36: 36-item Short Form Health Survey
SLS I: Scleroderma Lung Study I
SSc-ILD: systemic sclerosis–interstitial lung disease
TLI: total lung involvement
WL: whole lung
ZM: zone of maximal involvement
