# Supplementary figures and images for: Predictors of lung function decline in scleroderma-related interstitial lung disease based on high-resolution computed tomography: implications for cohort enrichment in systemic sclerosis–associated interstitial lung disease trials
Source: Arthritis Res Ther. 2015 Dec 23;17:372. doi: 10.1186/s13075-015-0872-2 (PMC4718035; doi:10.1186/s13075-015-0872-2)

## Slide 1
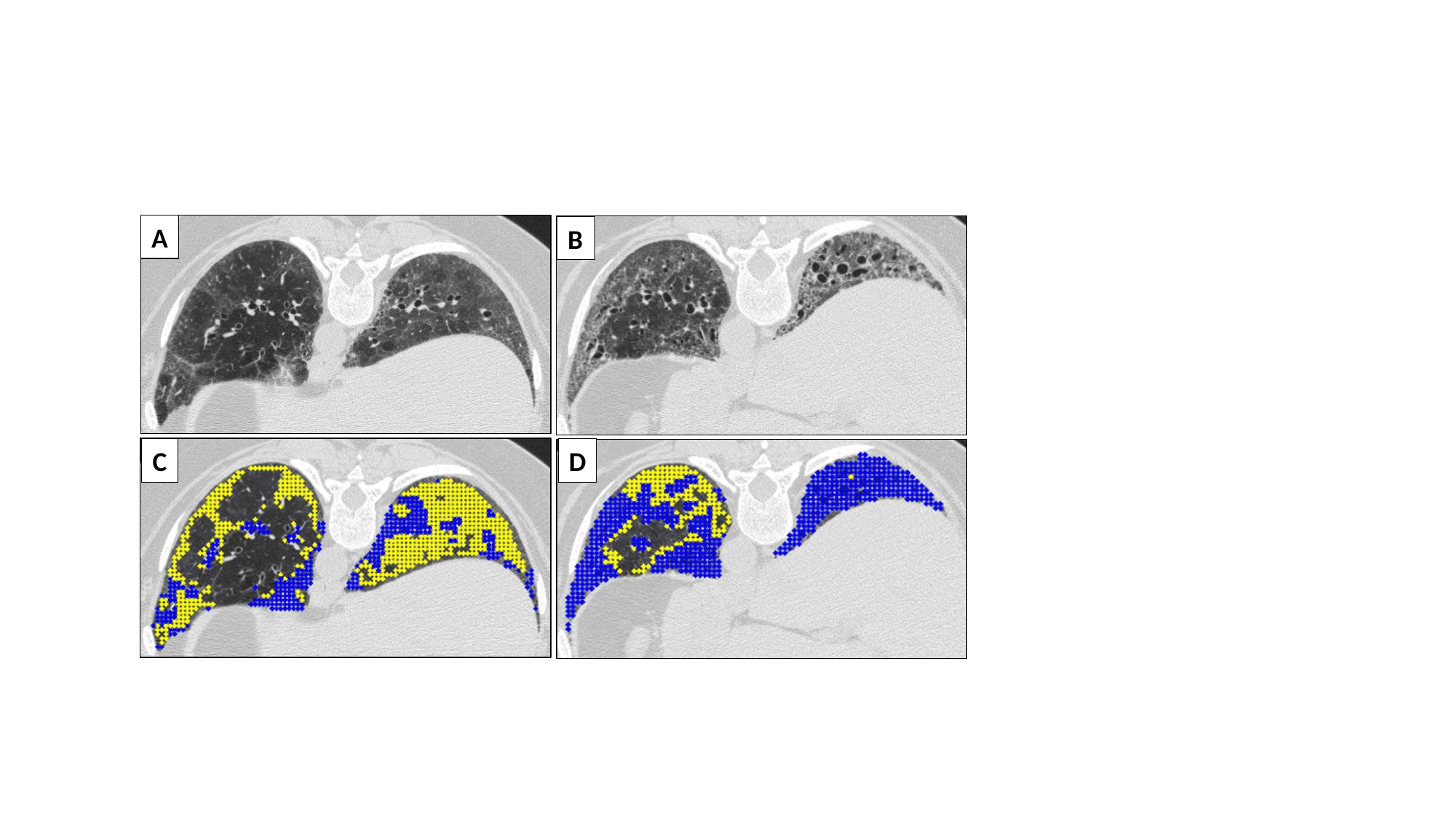

A
B
C
D

Supplement: Additional file 1: Figure S1. — Representative data from a subject at baseline and 12 months in the SLS I. Subject was 61 years old with baseline and 12-month HRCT. Goh and Wells unadjusted stratification >20 (a and b). In whole lung, quantitative lung fibrosis scores (blue dots) are 9.71 % at baseline (c) and 34.25 % at 12 months (d); quantitative ground glass (yellow dots) are 18.38 % at baseline and 25.8 % at 12 months, and QILD score are 38.09 % at baseline and 60.05 % at 12-month follow-up. (PPTX 945 kb) [file 13075_2015_872_MOESM1_ESM.pptx]
