# Supplementary material for: Predictors of lung function decline in scleroderma-related interstitial lung disease based on high-resolution computed tomography: implications for cohort enrichment in systemic sclerosis–associated interstitial lung disease trials
Source: Arthritis Res Ther. 2015 Dec 23;17:372. doi: 10.1186/s13075-015-0872-2 (PMC4718035; doi:10.1186/s13075-015-0872-2)
Supplement: Additional file 2: Table S1. — Relative decline in FVC % predicted (compared with baseline) over 12 months (DOCX 15 kb) [file 13075_2015_872_MOESM2_ESM.docx]

**Additional file 2: Table S1: Relative decline in the FVC% predicted (compared to baseline) over 12-months**

|  | Placebo group | | | Cyclophosphamide group | | | |
| --- | --- | --- | --- | --- | --- | --- | --- |
|  | N | Relative decline in FVC (%) from baseline Mean (SD) | P-value | N | | Relative decline in FVC (%) from baseline, Mean (SD) | P-value |
| Visual Max Fibrosis score | | | | | | | |
| 0 – 25% | 19 | 1.1 (14.2) | 0.01 | | 13 | -5.3 (9.9) | 0.03 |
| 26 – 100% | 29 | -9.5 (12.5) |  |  | 30 | 2.0 (10.3) |  |
| Goh / Wells Criteria | | | | | | | |
| <20% | 22 | -1.2 (15.6) | 0.08 | | 15 | -1.2 (10.3) | 0.63 |
| >20% | 25 | -8.6 (12.1) |  |  | 30 | 0.5 (11.8) |  |
| Goh / Wells Criteria with Indeterminate results on HRCT (10% to 30%) | | | | | | | |
| Limited disease | 8 | 5.1 (18) | 0.09 | | 12 | -1.4 (8.3) | 0.64 |
| Extensive disease | 39 | -7.5 (12.6) |  |  | 32 | 0.2 (12.3) |  |
| QILD WL [Whole lung] | | | | | | | |
| <20% | 10 | -0.03 (8.0) | 0.07 | | 5 | -11.2 (15) | 0.14 |
| >20% | 37 | -6.8 (15.3) |  |  | 38 | 1.2 (9.2) |  |
| QILD ZM [Zone of maximal involvement] | | | | | | | |
| <25% | 2 | 6.0 (9.1) | 0.30 | | 2 | -16.0 (0.7) | <0.01 |
| >25% | 45 | -5.8 (14.3) |  |  | 41 | 0.53 (10.2) |  |
| QLF WL | | | | | | | |
| <20% | 42 | -4.1 (13.8) | 0.18 | | 39 | -0.7 (10.3) | 0.55 |
| >20% | 5 | -15.4 (15.4) |  |  | 4 | 4.2 (14.3) |  |
| QLF ZM | | | | | | | |
| <25% | 27 | -1.3 (15.1) | 0.02 | | 24 | -2.0 (10.5) | 0.23 |
| >25% | 20 | -10.8 (11.2) |  |  | 19 | 1.9 (10.5) |  |

QILD WL= QILD whole lung, QILD ZM QILD= zone of maximal involvement, QLF WL= QLF whole lung, QLF ZM= QLF zone of maximal involvement
